# Observation of the Chiral and Achiral Hexatic Phases of Self-assembled Micellar polymers

**DOI:** 10.1038/srep32313

**Published:** 2016-08-31

**Authors:** Antara Pal, Md. Arif Kamal, V. A. Raghunathan

**Affiliations:** 1 Raman Research Institute, C V Raman Avenue, Bangalore 560 080, India

## Abstract

We report the discovery of a thermodynamically stable line hexatic (N + 6) phase in a three-dimensional (3D) system made up of self-assembled *polymer-like* micelles of amphiphilic molecules. The experimentally observed phase transition sequence *nematic (N*) 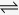 *N* + *6* 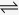 *two-dimensional hexagonal (2D*-*H*) is in good agreement with the theoretical predictions. Further, the present study also brings to light the effect of chirality on the N + 6 phase. In the chiral N + 6 phase the bond-orientational order within each “polymer” bundle is found to be twisted about an axis parallel to the average polymer direction. This structure is consistent with the theoretically envisaged Moiré state, thereby providing the first experimental demonstration of the Moiré structure. In addition to confirming the predictions of fundamental theories of two-dimensional melting, these results are relevant in a variety of situations in chemistry, physics and biology, where parallel packing of polymer-like objects are encountered.

The occurrence of the hexatic phase was first proposed in the context of melting of two-dimensional (2D) crystals, as an intermediate phase between the crystalline and liquid phases^1^,^2^,^3^. The hexatic phase is characterized by short-range translational order (TO) and long-range bond-orientational order (BO) (Fig. 1). Off all the theories that have been proposed so far to describe the melting of two-dimensional (2D) crystals, the one proposed by Kosterlitz-Thouless-Halperin-Nelson-Young (KTHNY theory of 2D melting)^1^,^4^,^5^ is probably the most appealing. The KTHNY theory proposes that the transitions between the crystalline and liquid phases are mediated by thermally activated topological defects. The 2D-crystal → hexatic transition is driven by the proliferation of free edge dislocations that destroy the long-range TO in the system, while the hexatic → liquid transition results from the loss of long-range BO due to thermally induced free dislocations.

Unlike their 2D counterparts, three-dimensional (3D) crystals melt directly into the liquid phase via a first-order transition. The exception however being 3D crystals made up of oriented polymer-like constituents where the possible presence of an intermediate hexatic phase has been theoretically demonstrated^6^. These systems are characterized by the long-range orientational ordering of the polymers along a direction specified by the nematic director 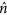. Examples of such systems include magnetic flux lines in type II superconductors, solutions of rigid or semi-rigid polymers and columnar liquid crystalline phases. As the temperature increases, order in the plane normal to 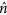 can go from crystalline to liquid-like through an intermediate hexatic region. The hexatic phase in these systems is referred to as the *line-hexatic* or N + 6 phase since nematic order coexists with BO in the plane normal to 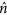
6. The N + 6 phase is thus expected to occur in between a nematic (N) phase, where TO in the plane normal to 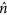 is short-range, and a 2D-hexagonal (2D-H) phase that characterizes 2D crystalline order in this plane.

Although the N + 6 phase has been theoretically predicted to occur as an intermediate phase between the N and the 2D-H phases, this is not the case in the two systems where this phase has been experimentally reported until now. In both these systems the N + 6 phase arises due to packing frustrations imposed by the shape of the individual constituents, and occurs close to their highest concentration compatible with 2D packing.

Occurrence of the N + 6 phase in 3D systems was first experimentally reported in dense aqueous solutions of long DNA fragments^7^,^8^, where its formation was attributed to geometric frustration arising from angular constraints imposed by the inter-DNA interaction potential, due to the double helical structure of DNA. As the concentration of DNA is increased further, the hexatic phase transforms into a crystalline phase with an orthorhombic unit cell through a distorted hexatic phase^9^. Contrary to theoretical predictions, molecular chirality was found to have no influence on the structure of the N + 6 phase in DNA solutions. This observation was rationalized in terms of either boundary effects that prevent a twist in BO^10^ or the coupling between translational and rotational motions of DNA which can lead to the overwinding of individual molecules themselves, rather than a twist in the BO^11^.

In the other instance, occurrence of the N + 6 phase was reported in dense suspensions of thin hard colloidal discs that stack into long columns^12^. This system exhibits a N → 2D-H → N + 6 phase sequence on increasing the particle density. Manifestation of the N + 6 phase at higher densities was attributed to geometric frustration arising from size polydispersity of the particles, which hinders long-range translational ordering of the columns. Thus quenched disorder plays an important role in stabilizing the line hexatic phase in these two systems^13^.

Influence of molecular chirality on the structure of the N + 6 phase has also been theoretically investigated^14^,^15^,^16^. These studies propose two possible structures for the chiral N + 6 phase. One of the structures proposed is a close analogue of the twist-grain boundary (TGB) phase observed in layered smectic liquid crystals^3^,^17^,^18^,^19^. In this structure the system breaks up into blocks with uniform orientation of 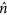 within each block. Adjacent blocks are rotated by a finite angle, leading to a structure where the twist axis is normal to 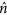. The second proposed structure is the *Moiré phase*, consisting of braided bundles of polymers, with BO in each bundle twisted about an axis parallel to the average polymer direction; in this phase the twist axis is parallel to 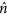. However, the structure of the chiral N + 6 phase still awaits experimental verification.

In this article we report the discovery of (a) a *thermodynamically stable* line-hexatic phase formed by self-assembled polymer-like micelles made out of a mixture of an amphiphile, sodium dodecylsulphate (SDS), and organic salt, p-toluidine hydrochloride (PTHC) (see Material and Methods section for a detailed discussion); (b) chiral analogue of N + 6 phase; and (c) the observation of the theoretically predicted N 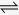 N + 6 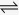 2D-H phase transition sequence as a function of temperature. Structure of the chiral N + 6 phase is found to be consistent with the theoretically predicted Moiré state^14^.

## Results and Discussions

**Figure 2:**
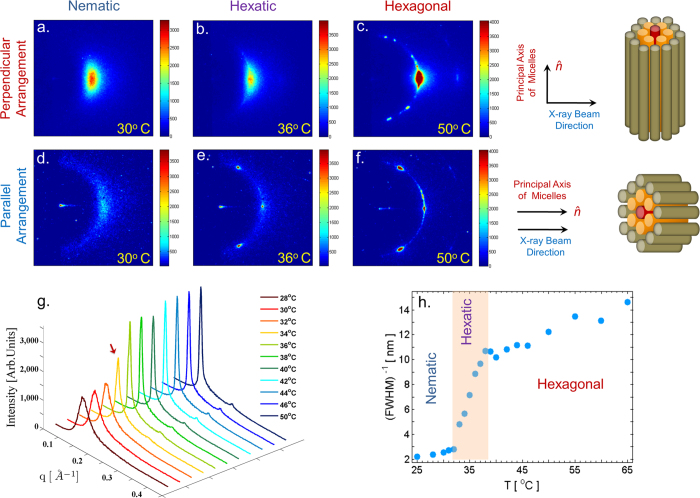
SAXS patterns of the achiral system in the *perpendicular* (X-ray beam is incident normal to 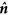; 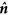 is parallel to z-axis;*q*_*z*_ is along the vertical) and *parallel* (X-ray beam is incident parallel to 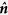; 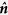 being parallel to z-axis; *q*_*z*_ normal to the plane of the diffraction pattern) geometries. Respective alignment schemes are shown on the upper right corner. (**a**,**d**) *Nematic*, N; (**b**,**e**) *Line-Hexatic*, N + 6; (**c**,**f**) *Hexagonal*, 2D-H. In the parallel geometry, long range Bond-orientational Order (BO) in the N + 6 and 2D-H phases gives rise to *three* sharp spots at an angular separation of 60°. (**g**) Intensity profiles as a function of q obtained at different temperatures; Red arrow indicates the onset of the N + 6 phase. (**h**) Variation of the inverse of the *full width at half maximum* (FWHM) of the diffraction peaks with temperature reveals a continuous increase in the correlation length (*ξ*) with increasing temperature. Orange band highlights the N + 6 region. Only half the pattern is visible due to the Kratky optics used.

Small-angle x-ray scattering (SAXS) patterns obtained using samples in the *perpendicular geometry*, where the incident x-ray beam is normal to 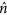 (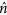 being oriented along z-axis), at different temperatures are shown in Fig. 2(a–c). Throughout the temperature range studied, the diffraction peak is found along *q*_⊥_, indicating that the cylindrical micelles have long-range orientational order along z. The position of the peak gives the average lateral separation between the micelles, while its width is inversely proportional to the translational correlation length *ξ* in the medium. The intensity profiles at different temperatures (Fig. 2g), reveal that the peak width gradually decreases with increasing temperature as shown in Fig. 2h. *ξ* is found to be ~1 nm in the N phase and ~10 nm in the 2D-H phase. Surprisingly instead of showing an abrupt jump, as is expected in case of a first-order N → 2D-H transition, *ξ* varies smoothly with temperature over a range of ~7 °C between these limiting values, thereby providing a first indication towards the possible presence of either an intermediate phase between the N and 2D-H phases or a coexistence of these two phases within this temperature range. Values of *ξ* obtained in the 2D-H phase are limited by the domain size in the sample, and not by the instrumental resolution. SAXS patterns of the high temperature phase show two sharp peaks, whose spacings are in the ratio 1: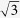, consistent with a 2D-H structure (Fig. 2c,g).

**Figure 3:**
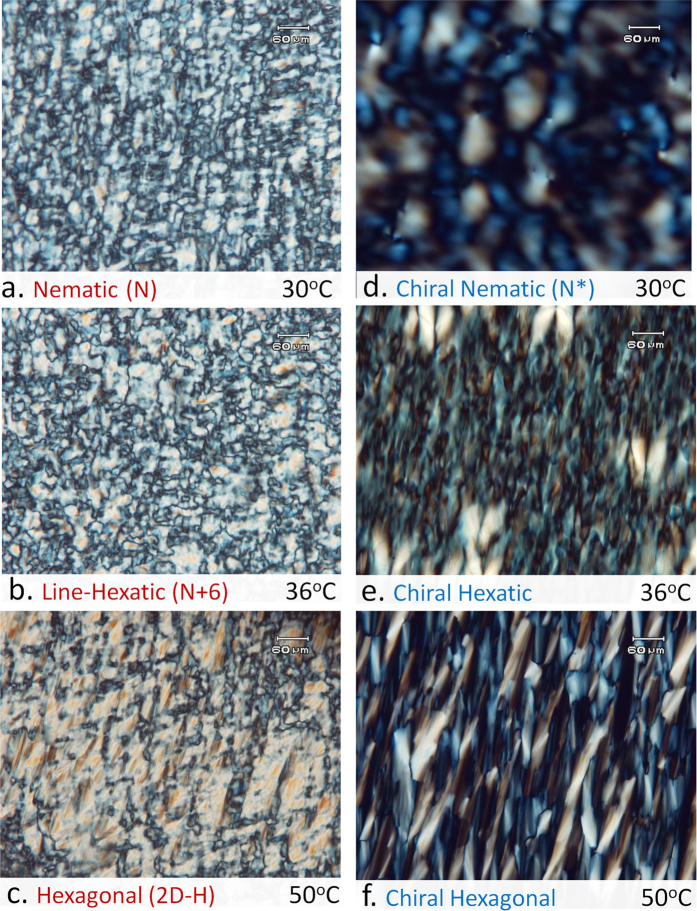
Typical polarizing optical microscope (POM) textures of the achiral (*left* column) and chiral (*right* column) systems. Textures of the Nematic and Line-Hexatic phases in the ***achiral*** system are indistinguishable in contrast they are quite different in the ***chiral*** system. Scale bar: 60 *μ*m.

In order to check the possible coexistence between the N and the 2D-H phases over the intermediate temperature range, the system was examined under a polarizing optical microscope (POM). Micrographs obtained at different temperatures are shown in Fig. 3(a–c). At low temperatures one observes thread-like *schlieren texture* in the POM micrograph which is characteristic of the N phase (Fig. 3a). On heating, the POM texture remains virtually unaltered till around 40 °C, beyond which sharper features appear abruptly, indicating a phase transition. However, in the intermediate temperature range (Fig. 3(b)), no indication of any coexistence between the two phases is found. This suggests that the gradual variation of *ξ* with temperature (Fig. 2(g)) can be attributed to the formation of an intermediate phase with longer translational correlation length as compared to the low temperature N-phase. Similar gradual variation of the correlation length across the isotropic - hexatic transition has also been reported in smectic liquid crystals^20^.

To ascertain the structure of the intermediate phase we have probed the nature of the BO in a plane normal to 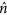. The system was investigated in the *parallel geometry* with the incident x-ray beam parallel to 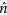. SAXS patterns obtained at three different temperatures in this geometry are shown in Fig. 2(d–f). Absence of long-range BO in the N phase at 30 °C leads to a broad ring in the *q*_⊥_ plane. In contrast, long-range BO in the high temperature (50 °C) 2D-H phase gives rise to six sharp spots in the *q*_⊥_ plane (Fig. 2f). Interestingly, six distinct spots are also observed in the pattern (Fig. 2e) corresponding to an intermediate temperature of 36 °C. This indicates that BO is long-ranged in the plane normal to 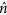 thereby confirming the intermediate phase to be the N + 6 phase.

**Figure 4:**
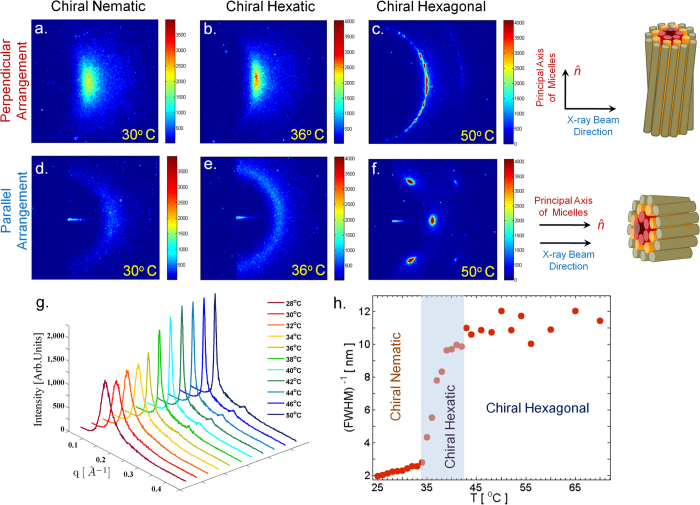
SAXS patterns of the chiral system in the *perpendicular* and *parallel* geometries (as defined in Fig. 2). (**a**,**d**) Chiral Nematic, N*; (**b**,**e**) Chiral Hexatic; (**c**,**f**) Chiral Hexagonal. In the parallel geometry, long range BO in the chiral hexagonal phase gives rise to *three* sharp spots at an angular separation of 60°, whereas in the chiral hexatic phase, BO twists about an axis parallel to 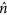, and manifests as a broad ring in the q_⊥_ plane. (**g**) Intensity profiles as a function of *q* obtained at different temperatures. (**h**) Variation of the inverse of FWHM of the diffraction peaks with temperature reveals a continuous increase in *ξ* with increasing temperature. Blue band highlights the chiral hexatic region.

Presence of an intermediate phase is further confirmed by POM observations of chiral samples, prepared by doping the achiral system with 1 wt.% of cholesterol (Fig. 3d–f). At low temperatures (Fig. 3d) the thread-like texture of the nematic phase is seen in freshly prepared samples, as in the achiral system. *Finger-print* texture characteristic of chiral nematic (N*) phase develops only after about 10 hours, presumably due to the very high viscosity of these samples. Two transitions, indicated by abrupt changes in the texture, are seen at around 33 °C and 40 °C on heating; which correspond closely to the boundaries of the region highlighted in Fig. 4h, where *ξ* varies strongly with temperature. These transitions are reversible on cooling. SAXS peak width of the chiral sample shows identical temperature dependence as that of the achiral sample (Fig. 2h). Observation of two clear transitions in the chiral samples again rules out the possibility that the observed temperature dependence of the peak width is due to the coexistence of the N and 2D-H phases. It also suggests that (a) the structure of the intermediate phase is different in the chiral system and (b) that the kinetics of its formation from the N* and 2D-H phases is very fast in spite of the high viscosity of the samples.

SAXS patterns of the chiral system in the perpendicular geometry (Fig. 4(a–c)) are identical to those of the achiral system (Fig. 2(a–c)), showing that the cylindrical micelles are oriented with their axes along z in all the three phases. In the case of the chiral nematic (N*) phase, molecular chirality is expected to result in a spontaneous twisting of the director field about an axis normal to 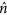. However, the sample preparation protocol used was found to result in well-aligned samples, with uniform orientation of 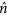. The alignment deteriorated only after keeping the samples for many hours. This is consistent with POM observations mentioned earlier, where the *finger-print* texture characteristic of the N* phase develops only after many hours in these highly viscous samples. As a result, orientation of the micelles is preserved in all the three phases during SAXS data collection from freshly prepared samples. This opportune feature makes it possible to probe BO in the plane normal to 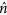 even in the chiral system.

**Figure 5:**
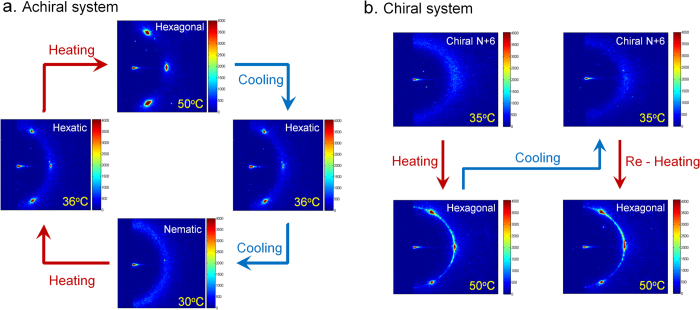
(**a**) SAXS patterns of the ***achiral*** nematic (N), Hexatic (N + 6) and Hexagonal (2D-H) phases in the parallel geometry, (x-ray beam incident *parallel* to 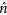) collected during temperature – cycling between the two phases. Both the N + 6 and the 2D-H phases show very similar patterns having 6-fold rotational symmetry, whereas the N phase shows a ring. (**b**) SAXS patterns of the ***chiral*** N + 6 (top) and 2D-H phases (bottom) in the *parallel* geometry, collected during temperature-cycling between the two phases. Note that the spots with 6-fold rotational symmetry seen in the 2D-H phase are replaced by a ring of scattering in the chiral N + 6 phase.

Diffraction patterns of the N* and chiral 2D-H phases in the parallel geometry (Fig. 4(d,f)) are also identical to those of the achiral system (Fig. 2(d,f)), indicating the absence and presence of long-range BO in these two phases, respectively, as expected. However, SAXS pattern of the chiral N + 6 phase in the parallel geometry is different (Fig. 4(e)), with the appearance of a ring in the *q*_⊥_ plane, instead of the six spots seen in the achiral system (Fig. 4(e)). In order to check if this change in the diffraction pattern is due to degradation of the sample alignment, we have repeatedly cycled the samples between the chiral N + 6 and the 2D-H phases and collected their SAXS patterns which are shown in Fig. 5(a,b). A well-aligned pattern with six-fold symmetry is obtained each time the sample is heated to the 2D-H phase, but a ring is obtained on cooling down to the chiral N + 6 phase, confirming that the appearance of a ring in the diffraction pattern of the chiral N + 6 phase is not due to the loss of alignment of the samples.

SAXS pattern of the chiral N + 6 phase is consistent with the Moiré structure proposed for this phase^14^,^15^, which consists of bundles of polymers, with the axes of the bundles oriented along the average direction of orientation of the micelles. Within each bundle BO develops a twist, with the twist axis parallel to the axis of the bundle. This would lead to the smearing of the six-fold symmetric SAXS pattern into a ring in the *q*_⊥_ plane, as found in Fig. 4e. Moiré structure is also compatible with the fast kinetics of formation of this phase, since it does not involve large-scale reorganization of the micellar arrangement. Existence of the other possible structure, similar to TGB, can be ruled out in the present case, since it would lead to a SAXS pattern consisting of a ring in the *q*_⊥_ - *q*_*z*_ plane in the perpendicular geometry, instead of the spots observed at *q*_*z*_ = 0.

Unlike previous reports of the N + 6 phase, where transitions between the different phases are driven by concentration, phase transitions in the present system are driven by temperature, which makes it possible to confirm the thermodynamic stability of these phases. Thermodynamic stability of the observed N, N + 6 and 2D-H phases and their chiral analogues was confirmed by repeatedly cycling the samples across these phases as a function of temperature. Diffraction patterns obtained in the parallel geometry, Fig. 5, clearly indicate that in all our experiments, we consistently recover all of these phases. Further, in contrast to the two earlier systems, the N + 6 phase in the present system occurs in between the N and 2D-H phases, as theoretically predicted. The observed phase sequence is, however, ***reversed***, in a sense that the system melts on cooling and not on heating.

**Figure 6:**
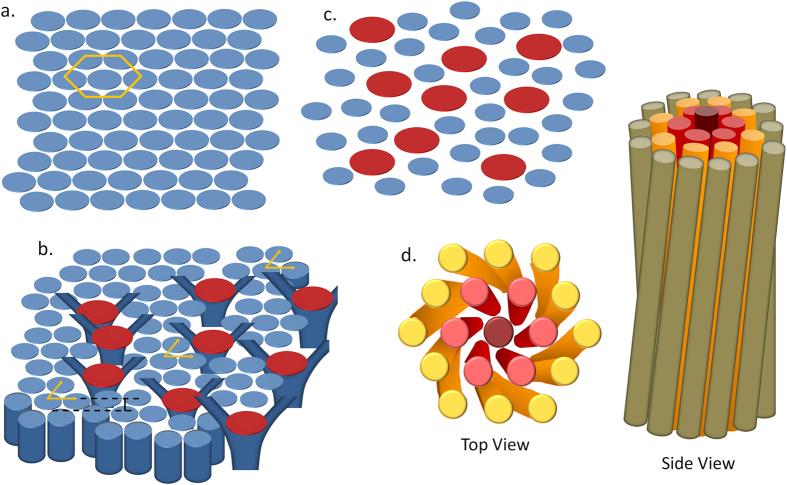
Schematic representation of the order in the plane normal to the micellar axis in the (**a**) crystalline, (**b**) N + 6 and (**c**) N phases. Larger (*red*) discs in (**b**,**c**) correspond to the branch points in the micelles whereas the smaller (*blue*) ones represent the non-branched ones. Increasing the number of branch points introduces packing constraints leading to the formation of the N + 6 phase. Side and the top views of a braided bundle making up the *Moiré* phase of the chiral N + 6 phase are shown in (**d**).

Polymer-like micelles typically form in ionic amphiphile-water systems in the presence of a salt (which provides counterions that bind to the micelles^21^,^22^) and are known to develop branches at higher salt concentrations^23^. In some of these systems, the counterions have been reported to desorb on increasing the temperature^24^. In the absence of branch points, the cross-section of the hexagonal phase formed by these micelles in the plane normal to the micellar axis will show a regular hexagonal lattice of mono-disperse discs (Fig. 6). In this cross-section, the branch points will appear as larger discs (Fig. 6). This situation is reminiscent of 2D packing of spheres of two different sizes, where it is known that for certain range of values of the two radii, a *crystal* → *hexatic* → *isotropic* transition sequence can be observed as the density of the larger sphere is increased^25^,^26^. In the present system, branch points play the role of the larger spheres. The observed N → N + 6 → 2D-H transition sequence with increasing temperature is consistent with this picture, since the concentration of branch points can decrease with increasing temperature due to the desorption of the counterions.

## Conclusion

In conclusion, we furnish in this article the first experimental validation of the theoretical prediction^6^ regarding the occurrence of a line hexatic phase in between the nematic and hexagonal phases, by demonstrating its existence in self-assembled polymer-like micelles of amphiphilic molecules. The observed phase sequence is, however, ***reversed***, i.e. the system melts on cooling rather than heating. Unlike previous reports on the N + 6 phase, where transitions between the different phases are driven by *concentration*, in the present system transitions are driven by *temperature*, which enables us to confirm their thermodynamic stability. Further, our investigations on the effect of chirality on the line hexatic phase also provides a first experimental verification of the theoretical predictions on the structure of the chiral hexatic phase. We find that chirality leads to the twisting of BO consistent with the theoretically predicted *Moiré* structure^14^,^15^. Ordered line liquids, similar to the one studied here, also occur in many condensed matter systems, such as magnetic flux lines in superconductors, nematic polymers and electro-rheological and ferro fluids^2^. Further, close-packing of chiral fibers is ubiquitous in biology^27^. Braided bundles, similar to the ones making up the Moiré phase, have been observed in the case of actin filaments^28^,^29^. Therefore, we believe that the results of the present study are relevant in a variety of situations, in addition to confirming the predictions of fundamental theories of 2D melting.

## Methods

Sodium dodecylsulphate (SDS) (>99% purity) and p-toluidine hydrochloride (PTHC) (98% purity) were obtained from Sigma-Aldrich. Experiments were performed at a PTHC to SDS molar ratio of 0.1 ([*PTHC*]/[*SDS*] = 0.1) and the total surfactant weight fraction of 0.5 (SDS + PTHC)/(SDS + PTHC + water) = 0.5)^30^,^31^. Ternary solutions of desired concentration were prepared by adding deionized water to appropriate amounts of PTHC and SDS weighed into sample tubes. The sample tubes were then sealed and allowed to homogenize at 40 °C typically for two weeks.

The textural observations of the various phases were carried out using polarized light optical microscopy (Olympus BX50) provided with a heating or cooling stage (Mettler FP82HT) and a central processor (Mettler FP90). Appropriate amount of samples were taken between a glass microscope slide and a glass coverslip. They were then placed inside the temperature-controlled chamber (heating or cooling rate 1 °C/min) and observed between crossed polarizers. The temperature of each sample was varied from 25 to 60 °C.

SAXS experiments were carried out using a Hecus SAXS-Eye system, in conjunction with either a CCD detector or a position sensitive detector. Details about the experimental set up and data acquisition have been described quite elaborately elsewhere^32^. The instrumental resolution was 0.06 nm^−1^. Temperature stability was better than 0.1 °C. Samples for SAXS studies were taken in sealed glass capillaries. All the samples were initially aligned in the nematic phase and the alignment was found to be retained on heating to the higher temperature phases. Orientation of the average micellar axis, i.e. the nematic director 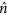, along the capillary axis was achieved by drawing the samples into 0.5 mm diameter capillaries. These samples were used in the perpendicular geometry, with the incident x-ray beam normal to 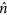, to probe the average orientation of the micellar axis in the medium. Orientation of 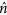 normal to the capillary axis was obtained by using two capillaries of different diameters in a Couette-cell-like arrangement. These samples were used to probe bond-orientational order in the medium in the plane normal to 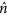. Capillaries were sealed subsequently to prevent evaporation of water. Both types of sample alignments were found to be preserved for many hours, much longer than the time required for SAXS measurements.

## Additional Information

**How to cite this article**: Pal, A. *et al*. Observation of the Chiral and Achiral Hexatic Phases of Self-assembled Micellar polymers. *Sci. Rep.* **6**, 32313; doi: 10.1038/srep32313 (2016).

## Figures and Tables

**Figure 1:**
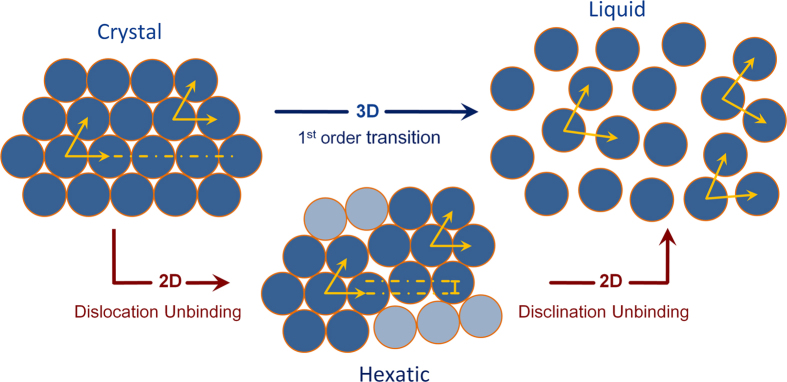
Schematic representation of the melting pathway of a crystalline phase into the liquid (isotropic) phase. Melting in 3D is a one step process (shown by the *blue* arrow), whereas in 2D it can be a two step process involving an intermediate hexatic phase as proposed in the KTHNY theory. In the crystalline phase, both the translational order (TO) as well as the bond orientational order (BO) are long ranged as indicated by the *yellow* arrows, whereas in the liquid phase both are short ranged. In the hexatic phase however, TO is short ranged but BO is long ranged.
